# High Cognitive Flexibility Learners Perform Better in Probabilistic Rule Learning

**DOI:** 10.3389/fpsyg.2020.00415

**Published:** 2020-03-13

**Authors:** Xia Feng, Garon Jesse Perceval, Wenfeng Feng, Chengzhi Feng

**Affiliations:** Department of Psychology, School of Education, Soochow University, Suzhou, China

**Keywords:** cognitive flexibility, rule learning, probability, switch cost, reward

## Abstract

Cognitive flexibility reflects the ability to switch quickly between tasks or stimulus sets, which is an important feature of human intelligence. Researchers have confirmed that this ability is related to the learners’ academic achievement, cognitive ability, and creativity development. The number-letter switching task is an effective tool for measuring cognitive flexibility. Previous studies have found that high flexibility individuals perform better in rule-based tasks such as the Iowa Gambling Task. It is not clear whether highly flexible learners have learning advantages when the rule tasks involve probabilistic cues. Using an inter-individual differences approach, we examined whether cognitive flexibility, as assessed by the number-letter task, is associated with the learning process of a probabilistic rule task. The results showed that the high flexibility group reached a higher level of rule acquisition, and the accuracy during the post-learning stage was significantly higher than the low flexibility group. These findings demonstrate that cognitive flexibility is associated with the performance after the rule acquisition during the probabilistic rule task. Future research should explore the internal process of learning differences between high and low flexibility learners by using other technologies across multiple modes.

## Introduction

As a core component of executive functioning (EF), cognitive flexibility has attracted much attention in psychological research. Research from various fields has investigated the internal mechanism underlying cognitive flexibility. Animal-based research has explored the underlying mechanisms of this function from an anatomical neurology perspective (Darby et al., 2018). Developmental psychologists focus on the training and growth of cognitive flexibility in children and adolescents (Dajani and Uddin, 2015). Studies of patients with neurological impairment also provide a window for exploring internal mechanisms (Lange et al., 2017). Despite extensive attention and research, there is still no clear common definition of cognitive flexibility, which can influence how this construct is operationalized in research (Müller et al., 2014). Based on the understanding that cognitive flexibility refers to “the ability of switching between tasks and stimulus sets in a quick and flexible manner” (Diamond, 2013; Müller et al., 2014), previous studies measured cognitive flexibility using scales or cognitive tasks (e.g., Wisconsin Card Sorting Test, WCST; task- switching paradigms) and researches have proved subjects with different levels of cognitive flexibility have different behavioral and neural characteristics (Müller et al., 2014). Although there is yet no clear conclusion about the mechanisms underlying cognitive flexibility, many researchers hold that cognitive flexibility is a prerequisite for many psychological functions, and it is one of the most important factors affecting intelligence and creativity (Diamond, 2013). For children, cognitive flexibility is a significant predictor of academic performance (Stad et al., 2018). High cognitive flexibility learners, including children (Lehto and Elorinne, 2003) and adults (Dong et al., 2016), usually show better performance on learning task, such as the Iowa Gambling task, which involves decision-making under uncertainly and has partly common neural mechanisms with rule learning (Hartstra et al., 2010).

Rule learning is based on stimulus patterns and feedback of behavioral outcomes to discover the relationship between operations and outcomes. Upon mastering the relationship, learners develop guidelines for subsequent behavioral choices, allowing them to further predict the corresponding results. This process enables an individual to recognize new information that expands upon existing knowledge. From the perspective of cognitive psychology, this process can be summarized as follows: the brain encodes stimuli, stimuli and feedback is used to construct rules, these rules are used to predict subsequent stimuli, and these rules are also applied to other similar stimuli (Dehaene et al., 2015). Hypothesis testing is at the core of rule learning (Klayman and Ha, 1989; Liu et al., 2015). During rule learning tasks, rule learning can enter the application stage smoothly if the hypothesis is successfully tested. If the hypothesis cannot explain the stimulus sequence, it must undergo further revision by the participant. This process will be repeated until the correct hypothesis is found or the experiment has ended. Successful hypothesis testing requires flexible switching among multiple possible hypotheses. High cognitive flexibility learners show better abstract induction, working memory, and feedback learning abilities during the Iowa Gambling task (Dong et al., 2016), which has some common neural basis of rule learning (Hartstra et al., 2010). We speculate that high-flexibility individuals may display more accurate and faster rule acquisition during rule learning as a result of their cognitive advantages.

In contrast to deterministic rule learning, there is no one-to-one matching relationship between cues and results in probabilistic rule learning. To use weather forecasting as an example, a “dark cloud” cue may result in “rain” in 70% of cases. Yet, in 30% of cases, the result is “cloudy.” Therefore, the cue “dark cloud” cannot be fixed to a certain attribute (i.e., “rain”), and the same reaction to “dark cloud” may be reinforced as “rain” or “cloudy.” It is impossible for learners to achieve complete error-free performance, and they eventually accept certain inevitable mistakes (Craig et al., 2011). This study aims to explore, for the first time, whether healthy adults with high cognitive flexibility show an advantage during a probabilistic rule learning task, just as in other rule-based learning tasks (i.e., Iowa Gambling Tasks; Dong et al., 2016). The WCST is perhaps the most widely used tool to measure cognitive flexibility in neuropsychology at present. However, compared to WCST, task-switching paradigms can provide a more pure measurement of cognitive flexibility by reducing the demand for working memory, classified learning and rule reasoning (Buchsbaum et al., 2005; Lange et al., 2018). This study uses the classical “number-letter task” to measure learners’ cognitive flexibility. Performance on the “number-letter task” (switch cost) will be used to divide participants into high and low cognitive flexibility groups, and their dynamic learning characteristics in different stage of probabilistic rule learning will be explored.

## Materials and Methods

### Participants

Three hundred and ten undergraduates from Soochow University completed the number-letter task. Data from 13 subjects was excluded for responding too quickly (RT below 100 ms), giving repeated responses, or misunderstanding the instructions. Data from 297 subjects (60 males) aged from 17 to 26 (*M* = 18.7, *SD* = 1.5) were used for further grouping. All subjects were right-handed, had normal or correct-to-normal vision, and no reported cognitive impairment. None of the subjects had participated in similar experiments. Participants were reimbursed according to their performance in the coin-searching task. All subjects had given written informed consent. The study protocol was approved by “the Ethical Committee of Soochow University.”

### Materials and Procedure

#### Number-Letter Task

In the classic number-letter task (Rogers and Monsell, 1995), a letter plus a number (e.g., 2U or M5) appears in a quadrant at the center of the screen. Letters are either vowels (A/E/I/U) or consonants (G/K/M/R) and numbers are either odd (3/5/7/9) or even (2/4/6/8). A letter and a number are randomly combined to form number-letter stimulus pairs. In the current study, the task consisted of practice and formal trials.

##### Practice trials

Letter, number and number-letter joint judgments were included in the practice trials. The sequence of letter and number judgments was balanced among subjects. For letter judgments, 32 trials (16 trials of consonants, half of them paired with an odd number) were included. Subjects were instructed to press ‘E’ or ‘I’ as quickly and accurately as possible to determine whether the letters were consonants or vowels. During the letter judgment trials, the stimulus pairs always appeared in the upper two quadrants. After an incorrect response, “ × ” would appear and the participants were instructed to re-press the correct key. The number judgment trials differed from the letter judgment trials in that the stimulus pairs always appeared in the two bottom quadrants and the subjects were required to determine whether the number was even or odd. In the combined number-letter trials, the stimulus pairs were presented clockwise one by one in each quadrant, and the number or letter was not the same as the previous one. For the stimulus pairs appearing in the upper two quadrants, letter judgments were needed, otherwise number judgments were required. Only when the accuracy rate was higher than 80% could participants enter the formal trials.

##### Formal experiment

The formal experiment consisted of 128 trials of combined number-letter judgments. “ × ” without chance of correction would appear after incorrect responses. When the stimulus pair jumped from the first quadrant to the fourth quadrant, the subjects needed to switch from letter judgment to number judgment accordingly. Similarly, when the stimulus pair jumped from the third to the second quadrant, the judgment should change from number to letter. We classified these as switching trials. When the stimulus pairs jumped from the fourth to the third quadrant or the second to the first quadrant, there was no need for task type switching. We classified these as non-switching trials.

The latency difference between switching and non-switching trials was regarded as the switch cost (the switch cost of latency = the average latency of the correct response in the switching trials – the average latency of the correct response in the non-switching trials). Switch cost was used to distinguish learners with high and low cognitive flexibility. High flexibility participants had a smaller switch cost, and the switch cost of low flexibility participants was greater. Participants that with switch cost scores in the upper 27^th^ percentile were included in the low flexibility group, subjects with scores in the lower 27^th^ percentile were included in the high flexibility group. Only these high and low flexibility groups completed the coin searching task.

#### Coin-Searching Task

**FIGURE 1:**
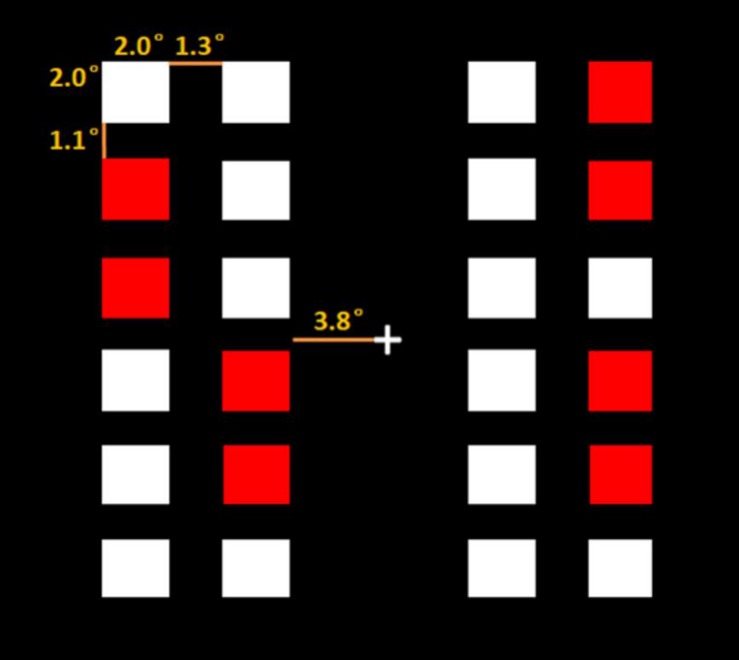
The visual angles of stimulation.

The coin-searching task is similar to that of Bellebaum and Daum (2008). E-prime 2.0 was used to program and run the task. The stimuli were presented on a 17′ computer monitor with a resolution of 1024 × 768 pixels. Each participant sat approximately 57 cm from the screen. Responses were recorded via ‘F’ and ‘J’ keys on a computer keyboard. There were 12 regular color blocks [RGB_red_ (255, 0, 0), RGB_white_ (255, 255, 255)] on the left and right sides of a black [RGB (0, 0, 0)] background. The visual angles of stimulation were shown in Figure 1.

The total number of red blocks was equal on both sides, with either 4 or 8 cases. The number of red blocks in the right column on both sides was either 0 or 2 or 4 or 6. If there was no red block in the right column of the selected side, the reward probability is 0. Similarly, the reward probability was 2/6 (1/3) for two red blocks, 4/6 (2/3) for four red blocks and 6/6 (1) for six red blocks of the selected side. The combinations of reward probabilities in single trial and the number of trials are shown in Table 1.

**TABLE 1:** The combinations of reward probabilities in a single trial and number of trials.

**Type of stimulus**		**Number of trials (Including left-right balance)**
**Reward probability of the right column for one side**	**Reward probability of the right column for the other side**	
0	1/3	120/540
0	2/3	90/540
1/3	2/3	240/540
1/3	1	90/540

Prior to beginning the task, the participants were told that: (1) Red and white blocks would appear on both sides of the fixation cross [The subjects were not informed that the total number of red blocks (4 or 8) was equal on both sides]; (2) A coin was hidden in one of the 12 colored blocks; (3) The task was to guess whether a coin was more likely to be hidden under a red block on the left (“F” key) or right (“J” key) side, and there was no need to judge a specific location for the coin; (4) There was a “rule” determining the reward and that correct identification and application of this rule would result in a greater reimbursement at study completion. Participants were not told beforehand the exact reimbursement amounts (correctly identifying rule: 50RMB, failure to identify rule: 40RMB).

**FIGURE 2:**
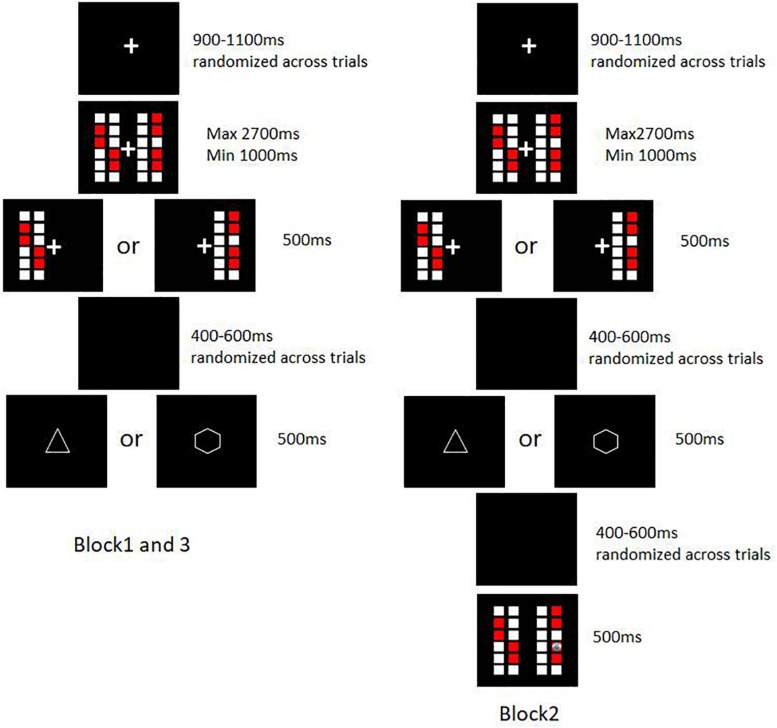
Schematic illustration of an exemplar trial in the coin-searching task.

The fixation point was presented with a random duration between 900 and 1100 ms. Then the color blocks were presented on two sides of the fixation cross. After the fixation point flashes, participants made a choice by pressing the “F” or “J” key with the left or right index finger respectively. The minimum reaction time was 1000 ms and the maximum was 2700 ms. The selected side would present for another 500 ms. After a 400—600 ms interval (black screen), feedback was presented for 500 ms. A triangle or a hexagon was represented to indicate reward or no reward respectively, which was balanced between subjects. There were three blocks, with 540 trials in total. Block 1 and Block 3 were identical. In Block 2 (trial 181–360), the participants were given additional feedback indicating the exact location of the coin: in the reward trials, the coin would appear under one of the red blocks in the right column of the selected side; and in the non-reward trials, the coin would appear under a white block in the right column of the selected side. The exact location of coins was determined at random. The procedure is shown in Figure 2. After finishing the experiment, subjects completed a questionnaire assessing rule identification success. In the questionnaire, subjects firstly described the rule he/she had found in as much detail as possible, and then evaluated his/her own confidence in the rules described before with one of six points (6-quite sure, 5-pretty sure, 4-a little sure, 3-a little not sure, 2-pretty not sure, 1-quite not sure).

The rule of the task is that the reward probability is determined by the ratio of red blocks in the right column of the chosen side. Choosing the side with a higher ratio of red blocks in the right column (i.e., a larger number of red blocks) will result in a higher likelihood of receiving a reward. In Figure 2, the reward probability is 1/3 for the left side and 2/3 for the right. Although there is a 1/3 probability of receiving no-reward upon choosing the right side, the right side is the correct choice since it has a higher chance of reward than the left side.

To control for potential left/right dominance effects, half of the participants were instructed to make their correct choice according to the comparison of reward probability in the two left columns of each side.

#### Data Analysis

For the number-letter task, participants were ranked according to their switch cost. The first 27% (smaller switch cost) of the participants were assigned to the high flexibility group, and the last 27% were assigned to the low group.

**FIGURE 3:**
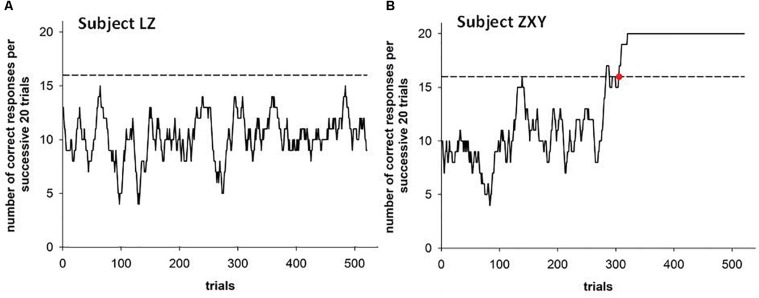
The learning curve of a typical rule non-learner **(A)** and a learner **(B)**.

In order to analyze the dynamic learning characteristics of probabilistic rule tasks, a window analysis with 20 window lengths and 1 step length was used. A stable performance criterion of ≥ 80% correct choices (≥ 16 correct responses within 20 successive trials) was considered successful task rule learning (learning baseline) (Bellebaum and Daum, 2008). For the subjects who learned the rule, the crossover point of the dynamic learning curve and the learning baseline (as shown in Figure 3B) was used as the key point to distinguish pre- and post-learning stages. If the subjects did not find any rule during the experiment, all responses were regarded as pre-learning in the subsequent analysis; similarly, if a participant learned the rule at the beginning of the experiment participant responses were regarded as post-learning only. A mixed analysis of variance (ANOVA) [2(high/low flexibility) × 4(probability pair) × 2(learning stage)] was adopted for the accuracy and latency scores of the coin-searching task. High/low cognitive flexibility was a between-subjects factor, probability pair (0-1/3, 0-2/3, 1/3-2/3, 1-1/3) and learning stage (pre-and post-learning stage) were within-subjects factors. Statistical analysis was performed using SPSS22.0.

## Results

### Overview of the Data

High and low cognitive flexibility groups were created according to switch cost scores on the number-letter task. Participants with a score less than 525.3 ms were assigned to the high flexibility group, and participants with a score greater than 1041.1 ms were assigned to the low flexibility group (Table 2). 39 (15 males, age_39_ = 20.2 ± 2.0) high flexibility and 37 (5 males, age_37_ = 19.3 ± 1.6) low flexibility participants were willing to participate further. Given that this study comprises part of the first author’s doctoral thesis, there is a difference between the total number of participants and participants assigned to the high and low cognitive flexibility groups.

**TABLE 2:** Switching cost (ms) for high and low cognitive flexibility groups.

	**Minimum**	**Maximum**	**M ± SD**	**Lower27^th^ percentile**	**Upper 27^th^ percentile**
All participants (297)	137.5	2085.3	813.4 ± 389.2	525.3	1041.1
High cognitive flexibility group (39)	137.5	517.1	357.3 ± 109.9		
Low cognitive flexibility group (37)	855.9	2069.6	1161.1 ± 285.5		

**FIGURE 4:**
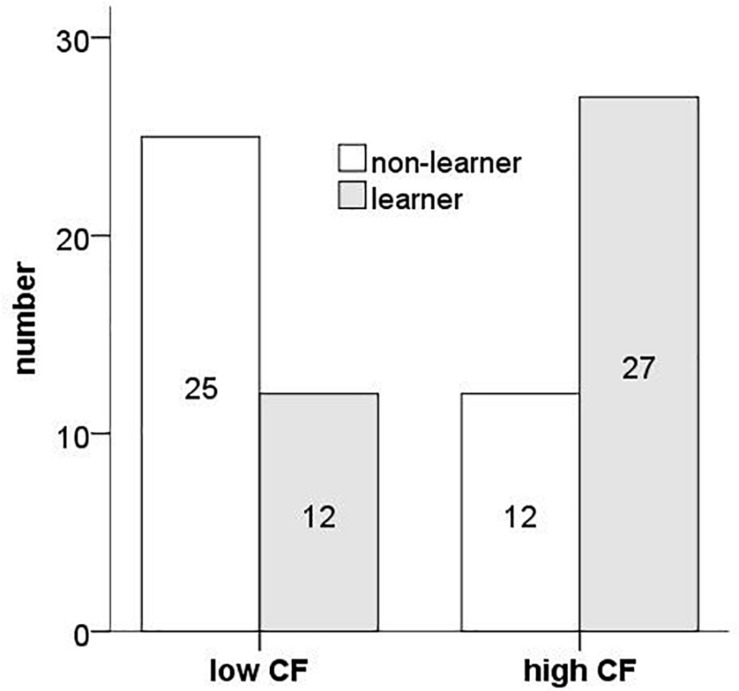
The number of rule discovers for high and low cognitive flexibility groups.

27 out of 39 (69.2%) participants in the high flexibility group and 12 out of 37 (44.4%) participants in the low flexibility group identified the correct rule (Figure 4). Pearson Chi-square test showed that the number of rule discoverers in the high group was significantly higher than that in low flexibility group [χ^2^ = 10.3, *df* = 1, *p* = 0.001].

**FIGURE 5:**
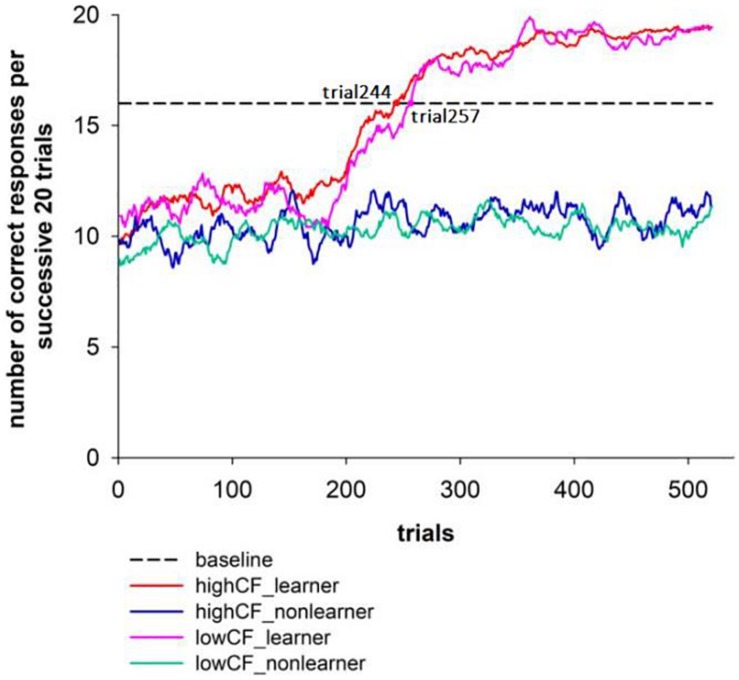
The average learning curves of the four groups.

In order to describe the dynamic learning process more closely, we plotted the learning curves of each participant. A typical rule learner and a non-learner are shown in Figure 3, and the average learning curves of the four groups (High CF – learner group: 27, and 3 out of 27 participants only had the post-learning stage since they had found the right rule with a few trials; high CF – non-learner group: 12; low CF – learner group: 12; low CF – non-learner: 25) are shown in Figure 5. The average learning point of all rule learners was 251 trials, which is the 71^st^ trial in block 2 (this block contains the specific feedback about coin position). Both the high and low flexibility groups reached their learning point in the second block (high CF group – 244, low CF group – 257).

Further, the confidence scores for the described rules of four groups (high CF – learner, high CF – non-learner, low CF – learner, low CF – non-leaner) and two groups (learner, non-learn)were compared. One-way analysis of variance for four groups showed that there was significant difference among four groups [*F*(3,72) = 3.108, *p* < 0.05], A Least-Squares Difference (LSD) test revealed high CF – learners’ confidence score [M ± SD = 4.6 ± 1.2] was significantly higher than that of low CF – non-learners [M ± SD = 3.6 ± 1.4]. Independent sample *t*-test showed the confidence score of learner group (*M* = 4.4, *SD* = 1.1) was significantly higher than non-learner group (*M* = 3.7, *SD* = 1.3) [*t*(76) = −2.761, *p* < 0.05, Cohen’s *d* = −0.626].

### Accuracy Analysis

**FIGURE 6:**
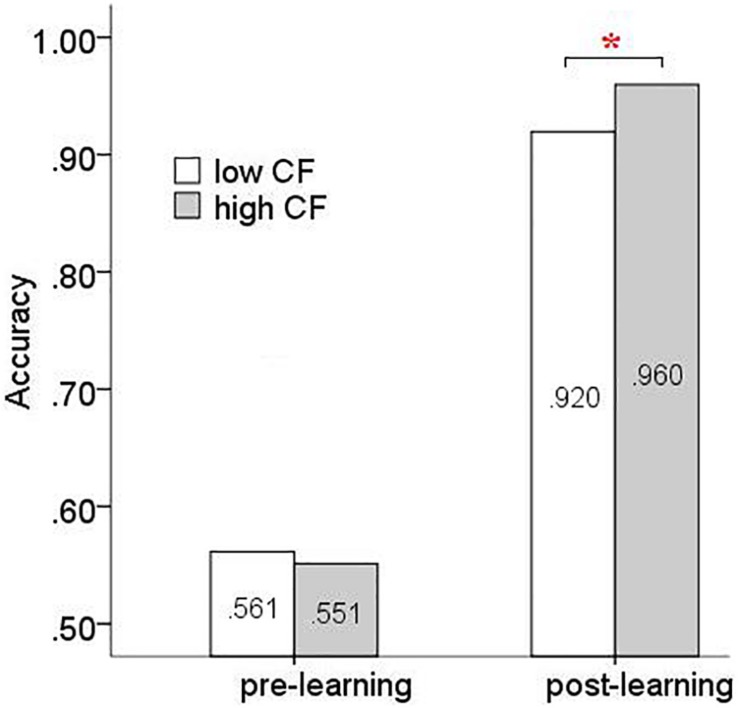
The accuracy of high and low CF groups during different learning stages (^∗^*p* < 0.05).

**FIGURE 7:**
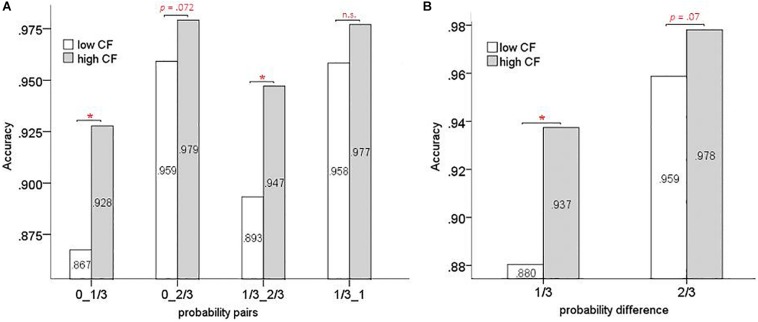
The accuracy of high and low CF groups during post-learning stage. **(A)** Accuracy in different probability pairs. **(B)** Accuracy in different probability differences (^∗^*p* < 0.05).

As mentioned above, the participants received feedback indicating reward or non-reward during the experiment. A correct response was recorded when participants chose the side with the greater number of red blocks in the right column (or left column, *n* = 38). Timed-out and unresponsive trials (1.16% of trials) were not included in the analysis. Accuracy scores were analyzed (accuracy = number of correct responses/total number of responses). A mixed measures ANOVA [2(high/low flexibility) × 4(probability pair) × 2(learning stage)] for participant accuracy scores showed a significant main effect of learning stage [*F*(1,34) = 709.728, *p* < 0.001, $\eta_{\text{p}}^{2}$ = 0.954] and probability pair [*F*(3,102) = 10.942, *p* < 0.001, $\eta_{\text{p}}^{2}$ = 0.243]. The interaction between learning stage and group was marginally significant [*F*(1,34) = 3.051, *p* = 0.090, $\eta_{\text{p}}^{2}$ = 0.082]. All other effects were not significant. An analysis of simple effects of high/low flexibility group and learning stage on accuracy showed a significant difference between the two groups after rule acquisition only [*F*(1,34) = 12.651, *p* < 0.05, $\eta_{\text{p}}^{2}$ = 0.271] (Figure 6). In order to further investigate the differences between groups after rule acquisition, a mixed measures ANOVA [4(probability pair) × 2(high/low flexibility)] was performed on the post-learning data. We observed a significant main effect of probability pair [*F*(3,111) = 19.889, *p* < 0.001, $\eta_{\text{p}}^{2}$ = 0.350] and group [*F*(1,37) = 11.662, *p* < 0.05, $\eta_{\text{p}}^{2}$ = 0.240]. However, the probability pair × high/low flexibility interaction was not significant (Figure 7A). Next we merged trials from the four probability pair conditions (0, 1/3; 0, 2/3; 1/3, 2/3; 1/3, 1) by averaging the accuracy of conditions with equal probability difference values. Two probability difference conditions were created: 1/3 probability difference (0, 1/3; 1/3, 2/3) and 2/3 probability difference (0, 2/3; 1/3, 1). A mixed measures ANOVA [2(probability difference) × 2(high/low flexibility)] showed a significant main effect of probability difference [*F*(1,37) = 48.914, *p* < 0.001, $\eta_{\text{p}}^{2}$ = 0.569] and high/low flexibility [*F*(1,37) = 11.662, *p* < 0.05, $\eta_{\text{p}}^{2}$ = 0.240]. A significant probability difference × high/low flexibility interaction was also observed [*F*(1,37) = 4.875, *p* < 0.05, $\eta_{\text{p}}^{2}$ = 0.116] (Figure 7B). Simple effects analysis showed there was a significant difference between high/low flexibility groups for the 1/3 probability difference [*F*(1,37) = 11.314, *p* < 0.05, $\eta_{\text{p}}^{2}$ = 0.234], and a marginally significant difference [*F*(1,37) = 3.482, *p* = 0.07, $\eta_{\text{p}}^{2}$ = 0.086] for the 2/3 probability difference.

### Latency Analysis

**FIGURE 8:**
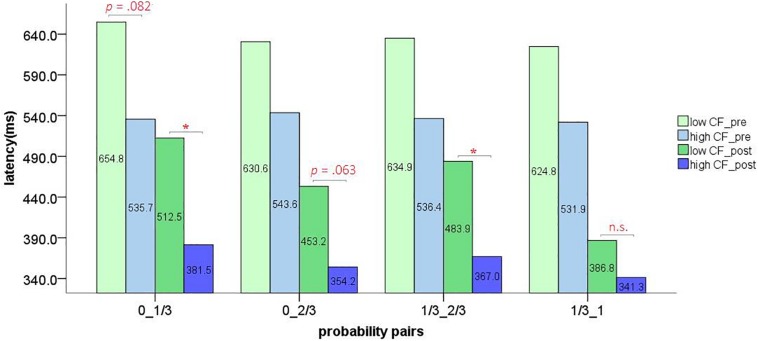
The latency of high and low cognitive flexibility groups for pre- and post-learning stages in different probability pairs (^∗^*p* < 0.05).

After deleting timed-out and unresponsive trials (1.16% of trials), a mixed measures ANOVA [2(learning stage) × 4(probability pair) × 2(high/low flexibility)] showed a significant main effect of learning stage [*F*(1,34) = 84.561, *p* < 0.001, $\eta_{\text{p}}^{2}$ = 0.713] and probability pair [*F*(3,102) = 10.030, *p* < 0.001, $\eta_{\text{p}}^{2}$ = 0.228]. A marginally significant main effect of group was also observed [*F*(1,34) = 3.395, *p* = 0.074, $\eta_{\text{p}}^{2}$ = 0.091]. Significant interactions between learning stage and probability pair [*F*(3,102) = 7.728, *p* < 0.001, $\eta_{\text{p}}^{2}$ = 0.185], and probability pair and high/low flexibility were observed [*F*(3,102) = 3.203, *p* < 0.05, $\eta_{\text{p}}^{2}$ = 0.086]. The three-way interaction was marginally significant [*F*(3,102) = 2.181, *p* = 0.095, $\eta_{\text{p}}^{2}$ = 0.060] and the interaction between learning stage and group was not significant [*F*(1,34) = 0.001, *p* = 0.973, $\eta_{\text{p}}^{2}$ = 0.000]. Simple effects analysis of the four probability pairs in the different learning stages showed

a significant difference among four probability pairs after rule learning [*F*(3,32) = 6.752, *p* < 0.05, $\eta_{\text{p}}^{2}$ = 0.388]. A Least-Squares Difference (LSD) test revealed significant differences between 0-1/3 and 1/3-1 probability pairs (*p* < 0.05) during post-learning. Simple effects analysis of the four probability pairs for the two flexibility groups showed that only the low flexibility group had significantly different latencies in the different probability pairs [*F*(3,32) = 5.571, *p* < 0.05, $\eta_{\text{p}}^{2}$ = 0.343]. The latency of high and low cognitive flexibility groups for pre- and post-learning stages in different probability pairs was shown in Figure 8. In order to further describe the reaction time differences within the low-flexibility group, a 2 × 4 repeated measures ANOVA [2(learning stage) × 4(probability pair)] was conducted on the data of the low group only. All main and interaction effects were significant [learning stage: *F*(1,11) = 24.340, *p* < 0.001, $\eta_{\text{p}}^{2}$ = 0.689; probability pair: *F*(3,33) = 6.244, *p* < 0.05, $\eta_{\text{p}}^{2}$ = 0.362; learning stage × probability pair interaction: *F*(3,33) = 3.623, *p* < 0.05, $\eta_{\text{p}}^{2}$ = 0.248]. Simple effects analysis showed a marginally significant difference among probability pairs after learning acquisition [*F*(3,9) = 3.250, *p* = 0.074, $\eta_{\text{p}}^{2}$ = 0.520]. Specifically, there were (marginally) significant differences between 0_1/3 and 0_2/3 (*p* = 0.089), 1/3_2/3 and 1/3_1 (*p* = 0.073), and 0_1/3 and 1/3_1 (*p* < 0.05). Additionally, the data of the 1/3 and 2/3 probability differences after rule acquisition were averaged respectively. A paired sample *t-*test showed a significant difference between 1/3 (*M* = 498.20 ms, *SD* = 176.54 ms) and 2/3 (*M* = 419.97 ms, *SD* = 135.34 ms) probability differences [*t*(11) = 3.406, *p* < 0.05, Cohen’s *d* = 0.24]. After successful rule learning, the low flexibility subjects responded faster to stimuli with a higher probability difference.

## Discussion

In the present study, learners were grouped into high and low cognitive flexibility groups based on their performance on the number-letter switching task. The learning characteristics of the two groups in a rule task with probabilistic cues were preliminarily explored. Behavioral data analysis showed that the differences between the two groups are mainly manifested in the following three points: (1) The high CF group showed a higher rate of rule acquisition, which partially verified our hypothesis that high cognitive flexibility learners would show more accurate rule acquisition during rule learning. However, the two groups showed very similar average rule acquisition points, the high flexibility group did not show faster rule acquisition as predicted. (2) The high flexibility group showed significantly higher accuracy than the low flexibility group after rule acquisition. (3) After rule learning, the low-flexibility group showed significantly different response latencies across the four probabilistic pairing conditions, while the high-flexibility group did not show such differences.

The rule acquisition speed of the high flexibility group was not faster than that of the low flexibility group, which may be related to the reward feedback provided in block 2. In order to reduce task difficulty, the exact coin position was shown to the learners during the second block (for reward feedback, the coin was shown under the red block on the dominant side; for non-reward feedback, the coin was shown under the white block). This feedback aided the subjects in identifying the existence of a “dominant side.” Combining this knowledge with the relationship between reward and red blocks, the subjects could identify the basis for response more easily. This design reduces the difficulty of the task (Bellebaum and Daum, 2008) which has been proved in previous study. However, we guess this design of exact coin position in block 2 may have weakened the inter-group differences of the high and low flexibility groups to some extent. It is possible to find the correct basis of reaction from the specific position of the coin for both high and low groups, and the average learning curves in Figure 5 also supported this. The cue of exact location of the coin provided participants with a shortcut to the task, which was open to both groups.

Even if the involvement of block design made the task became easier, the overall rule learning rate of the task in present study was 51.3% [(21 + 12)/(39 + 37)]. That is, only half of the participants identified the correct rule, which is lower compared with previous studies using this paradigm [66.7% (18/27)] (Bellebaum and Daum, 2008). This may be related to differences in participant cognitive flexibility levels: in Bellebaum’s study the level of flexibility was not a key factor of interest and so the overall level of cognitive flexibility in their sample is unknown. In the present study, 48.7% of subjects had low flexibility. This relatively large proportion of low flexibility subjects may explain the overall lower learning rate in our study.

After rule learning, the accuracy of the high flexibility group was higher than that of the low flexibility group for the 1/3 and 2/3 probability differences. Especially for the 1/3 probability difference, the advantage of the high flexibility group was more pronounced. This finding may indicate that the high-flexibility group used the response criterion correctly more frequently and that this group may have a greater mastery and confidence surrounding rule learning. Using the Iowa Gambling task, Dong et al. (2016) showed that people with high flexibility showed explicit knowledge of task rules whereas the low group did not, which is consistent with the higher response accuracy of the high flexibility group in this study. Their research also showed that the lower P300 amplitude of the low flexibility group in the stimulus selection evaluation stage might be due to the lower cognitive and abstract generalization abilities or working memory abilities of the low flexibility group. The present findings extend the advantage of the high cognitive flexibility group to probabilistic reward learning, that is, the high flexibility group could distinguish stimuli with little differences in probability more effectively at the later stage of learning. However, what are the differences in the internal learning processes that result in group differences? Müller et al. (2014) suggests that differences in cognitive flexibility among individuals is related to many factors, including gray matter volume of the right anterior insula, the functional connection between the bilateral anterior insula and the midcingulate cortex/supplementary motor areas, and the degree of impulsivity according to the Big Five personality traits. Different factors exert unique effects on cognitive flexibility. Research across multiple paradigms and using various methodologies (i.e., fMRI, EEG) is needed to further understand the mechanisms underlying cognitive flexibility.

There were significant differences among the probabilistic pairs for the low flexibility group. The differences were mainly due to faster responses to large probability differences (2/3) than to small probability differences (1/3) in the post-learning stage. However, the high flexibility group did not change significantly with the change of probability pairs. For the high flexibility group, although the number of red blocks on the left and right sides changed constantly according to the settings of the experimental conditions, high flexibility subjects may approach all conditions by applying a unified ‘framework,’ or response basis, with all conditions being parallel parts of that framework. For the low flexibility learners, they may not have employed such a framework. Their response basis may vary for each probability condition even after entering the post-learning stage. This may be evidenced by the low flexibility learners showing slower response latencies for the 1/3 probability difference condition that were not accompanied by accuracy levels comparable to that of the 2/3 condition (accuracy is significantly lower in the 1/3 probability difference for the low flexibility group). It is perhaps due to confusion about the response basis in the 1/3 probability difference condition that the latency under this condition is longer and the accuracy is lower. In fact, this condition appeared to be particularly difficult for the low flexibility subjects. This may be due to two reasons: (1) A greater number of learners in the low group did not identify the correct response criteria (rule) applicable to all conditions of the task during learning. (2) Because the probability attributes of the rules were not indicated before the task, and people generally tend to search for a simple ‘stimulus-response’ connection (i.e., correct response = reward), it may be a greater challenge for the low cognitive flexibility group to realize and accept a probability-based reward pattern.

Cognitive flexibility involves explicit and implicit forms of processing (Fujino et al., 2017), and the flexible goal achievement is not fully conscious (Custers and Aarts, 2010). Study has shown that there are different areas of brain activity in individuals with rationality-based explicit aspect of flexibility and experience-based implicit aspect of flexibility, and there is strong connection between them (Fujino et al., 2017). In present study, subjects were required to describe the founded rule after the task. Most of the subjects who had reached the acquisition level behaviorally could describe the correct rule while others report being unclear or not always following the same rule, not all the subjects who had learned the rule could grasp the rule consciously. However, the objective separation of implicit and explicit parts in probabilistic rule learning was not yet realized. Meanwhile, for the confidence of the rule, learner group had significant higher confidence score than non-learner group. When subjects were further divided into high CF – learner, high CF – non-learner, low CF – learner and low CF – non-learner group, only high CF – learners’ confidence score was significantly higher than that of low CF – non-learners. No matter high CF – learner vs. high CF – non-learner group or low CF – learner vs. low CF – non-learner group, the confidence scores were not significant. This was because although some subjects had not learned the rules objectively, they thought the rule had been found was correct and gave relatively high confidence scores.

As mentioned before, the study has reached some conclusions about differences between high and low CF learners in probabilistic rule learning. And, there are some issues not taken into account and deserve further attention. Firstly, the sample was imbalanced by sex, and most of the subjects were mainly women. The results of this study cannot exclude the effect of potential gender differences. Secondly, it is possible that the difference of two groups in acquisition speed of rule may be disguised as the setting of the task, which depends on the use of other probabilistic rule tasks for further investigation. Thirdly, the differences between groups were in behavioral, post-learning stage in detail. An unresolved issue concerns the question as to what is the cause of the differences before the rule is acquired. Further detailed analysis of the learning process using tools such as ERP will be helpful. Finally, in present study, the artificial 80% response accuracy rate was used as the cut-off point for the acquisition of rule, and the six-point scale also used to evaluate the learners’ certainty of rule after the completion of the coin-searching task. Whether there are some objective and implicit indicators of rule acquisition in probabilistic rule learning is an interesting point for further study.

## Conclusion

This study preliminarily confirms that there are significant differences in learning outcomes between high and low cognitive flexibility learners in probabilistic rule learning. In short, cognitive flexibility is associated with the performance after the rule acquisition during the probabilistic rule task. The deep-rooted reasons for these differences need to be further explored by using other experimental techniques.

## Data Availability Statement

The datasets generated for this study are available on request to the corresponding author.

## Ethics Statement

The study protocol was reviewed and approved by the Ethical Committee of Soochow University. The participants provided their written informed consent to participate in this study.

## Author Contributions

XF contributed to experimental design, data collection, data analysis, and manuscript writing. GP contributed to manuscript writing. WF contributed to data analysis and manuscript writing. CF contributed to experimental design.

## Conflict of Interest

The authors declare that the research was conducted in the absence of any commercial or financial relationships that could be construed as a potential conflict of interest.
